# Comparison of the KonRad IMRT and XiO treatment planning systems

**DOI:** 10.1120/jacmp.v9i3.2770

**Published:** 2008-07-14

**Authors:** Bodo Reitz, Moyed Miften

**Affiliations:** ^1^ Department of Radiation Oncology Allegheny General Hospital Allegheny Campus Pittsburgh Pennsylvania USA; ^2^ Drexel University College of Medicine Allegheny Campus Pittsburgh Pennsylvania USA

**Keywords:** TPS, IMRT, Segmentation, QA

## Abstract

Reducing the treatment time for IMRT patients is highly desirable. The objective of this work was to evaluate the new clinical Siemens KonRad inverse treatment planning system (TPS) and compare it to the CMS XiO TPS with special emphasis on the segmentation efficiency. For head and neck, liver and prostate cancer patients, step‐and‐shoot IMRT plans were designed using both CMS XiO and Siemens KonRad TPS. Number, direction and energy of beams used were the same in the plans from both systems for each treatment site. The plans were optimized to achieve the same clinical objectives concerning dose to the target volume and to the relevant organs‐at‐risk (OARs). The number of intensity levels were minimized until the clinical objectives could not be achieved anymore. Dose‐volume histograms (DVHs), mean and maximum doses were compared, as well as the number of beam segments and monitor units (MUs). The beams of each plan were delivered individually on a MapCheck device to verify the agreement between calculations and measurements to be less than 3%–3 mm distance‐to‐agreement. Plans optimized with KonRad resulted in fewer segments and lower number of MUs and therefore reduced delivery time on average by 28% or 3.6 min, while achieving similar dose distributions. CMS XiO plans exhibited a slightly steeper dose fall‐off outside the target volumes; however the difference was not clinically significant. DVHs to OARs were comparable. All calculated dose distributions passed the 3%–3 mm verification check.

PACS numbers: 87.55.D

## I. INTRODUCTION

Intensity modulated radiation therapy (IMRT) delivers increasingly conformal radiation fields to tumors with sharp dose‐gradients around the target volume while allowing better sparing of healthy tissue. In the static or step‐and‐shoot IMRT approach each IMRT field is composed of several overlaying segments with different weights and shapes, designed by the segmentation algorithm of the treatment planning system. The fields of each segment are shaped by the multi‐leaf collimator (MLC) and delivered sequentially. The MLC leaves stay stationary while the beam is turned on, and only move to reshape between the segments, while the beam is turned off. As compared to 3D conformal radiation therapy, the treatment time to deliver a similar dose is typically longer. Because the field is composed of smaller segments the total number of monitor units (MUs) is larger, and more importantly the time for moving the MLC leaves and subsequent need for record and verify cycles add a significant overhead to the total treatment time. Therefore, reducing the number of beam segments and MUs is a prerequisite for shortening the delivery time of step‐and‐shoot IMRT plans. Considering that cancer patients may have difficulties lying on the treatment couch for long periods of time during the radiation delivery, shortening the IMRT treatment time is highly desirable. It decreases the risk that patients involuntarily move during the radiation therapy delivery. It also minimizes the risk of decreased tumor cell killing potentially associated with fraction delivery times in the range of 15–45 min.^1^, ^4^ Several leaf sequencing algorithms have been discussed and compared in the literature.^5^, ^14^ Recently Siemens Medical Solutions released a new version of its KonRad IMRT (Siemens Medical Solutions, Concord, CA) treatment planning system,^(15)^ and received 510k approval for its clinical use in the U.S. from the Food and Drugs Administration (FDA). The objective of this work was to evaluate the clinical KonRad treatment planning system, with emphasis on the efficiency of the sequencing algorithm. The agreement between calculated and measured dose distributions was another objective of this work. The CMS XiO treatment planning system (CMS Inc., St. Louis, MO) was chosen as a baseline of all comparisons for this evaluation.

## II. METHODS

Six cancer patients, including two head and neck (H&N), one liver and three prostate cases, were selected for this study. For the first H&N case (HN1), a dose of 70 Cy in 35 fractions was prescribed to the primary planning target volume (${\text{PTV}}_{70}$). Additionally three other PTVs were treated with prescription doses of 65.8 Cy $\left({\text{PTV}}_{65.8}\right)$, 63 Gy (${\text{PTV}}_{63}$), and 57.8 Gy $\left({\text{PTV}}_{57.8}\right)$, respectively. The treatment prescription also included limitations on the mean or maximum doses to the optic chiasm $\left(D_{\text{max}}<45\;\text{Gy}\right)$, brainstem $\left(D_{\text{max}}<50\;\text{Gy}\right)$, oral cavity $\left(D_{\text{mean}}<40\;\text{Gy}\right)$, mandible $\left(D_{\text{mean}}<55\;\text{Gy}\right)$, left and right parotid $\left(D_{\text{mean}}<26\;\text{Gy}\;\text{and}<30\;\text{Gy},\;\text{respectively}\right)$, esophagus $\left(D_{\text{max}}<60\;\text{Gy}\right)$. For the second H&N case (HN2) three PTVs were defined with prescription doses of 62.4 Gy $\left({\text{PTV}}_{62.4}\right)$, 57.6 Gy $\left({\text{PTV}}_{57.6}\right)$ and 53.4 Gy $\left({\text{PTV}}_{53.4}\right)$, respectively.

For the liver case (LI), a single PTV was delineated and received a dose of 50.4 Gy in 28 fractions. Organs at risk (OARs) included in the radiation prescription were the lungs ($V_{20}<10\%)$, heart $\left(D_{\text{max}}<20\;\text{Gy}\right)$, liver, small bowel and kidneys. For the last three organs, dose‐volume histogram constraints were included in the prescription. For all three prostate cases (PR1, PR2, and PR3), the PTV received 77.4 Gy in 43 fractions. OARs for the prostate cases were rectum, bladder, and femoral heads.

The clinical IMRT treatment plans were designed with the established XiO treatment planning system (Version 4.33.02). These plans were used as a baseline for a comparison with plans generated by the new KonRad system (Version 2.2). All plans were designed as step‐and‐shoot IMRT plans for delivery on a Siemens Primus Accelerator with a 58 leaf MLC.

On both systems, plans were optimized to achieve the same clinical objectives concerning target dose, target coverage and sparing OARs. Beam energy, as well as number and direction of beams (five beams for the prostate and liver cases, seven beams for the H&N cases) were identical.

The cost or objective function F utilized by both treatment planning systems is based on physical constraints, namely dose and dose volume constraints – as opposed to biological constraints such as tumor control probability (TCP), normal tissue complication probability (NTCP), or equivalent uniform dose (EUD). The cost function has the general form:

(1) $$F=\sum_{k=1}^{K}F_{k}$$

with *K* the number of objectives and $F_{k}$ the subcost for an objective *k*. KonRad and XiO both utilize dose objectives for targets and OARs. The subcost function for a maximum dose constraint has the form:

(2) $$F_{k}=\frac{\nu_{k}}{M_{k}}\sum_{i=1}^{M_{k}}g_{k}(D_{i})\;with$$

(3) $$g_{k}(D_{i})=0\;for\;D_{i}<D_{0},$$

(4) $$g_{k}(D_{i})=m_{+}(D_{i}-D_{0})\;for\;D_{0}<D_{i}<D_{\max},$$

(5) $$g_{k}(D_{i})=m_{+}(D_{i}-D_{0})+{\left(D_{i}-D_{max}\right)}^{\Lambda }\;for\;D_{i}>D_{\max},$$

where $D_{0}$ is the desired or goal dose, $D_{\text{max}}$ the maximum dose, $V_{k}$ is the importance weight, $M_{k}$ is the number of voxels in the structure for objective ${\text{k, m}}_{+}$ is the linear penalty, and Λ is the penalty power. Similarly the objective for a minimum dose constraint has the form:

(6) $$F_{k}=\frac{\nu_{k}}{M_{k}}\sum_{i=1}^{M_{k}}g_{k}(D_{i})\;with$$

(7) $$g_{k}(D_{i})=0\;for\;D_{i}>D_{0},$$

(8) $$g_{k}(D_{i})=m\_\left(D_{i}-D_{0}\right)\;for\;D_{0}>D_{i}>D_{\max},$$

(9) $${g_{k}(D_{i})=m\_\left(D_{i}-D_{0}\right)+(D_{i}-D_{max})}^{\lambda }\;for\;D_{i}<D_{\max},$$

where $D_{0}$ is again the desired or goal dose, $D_{\text{min}}$ the minimum dose, $w_{k}$ the importance weight, m_‐_ the linear penalty and λ the penalty power. In addition to minimum and maximum dose constraints both treatment planning systems allow the use of dose volume constraints based on the work of Bortfeld et al.^(16)^

Although the general form of the objective function is similar in both systems, the implementation differs in some details. KonRad uses only a quadratic penalty; the linear penalty is not implemented. Therefore the parameters $m_{-}$, $m_{+}$, and $D_{0}$ are not used, and the penalty powers λ and Λ are set to 2. In XiO the penalty powers λ and Λ are user selectable with values between 2 and 5. The desired dose $D_{0}$ for OARs, and $1/2(D_{\text{max}}+D_{\text{min}})$ for target volumes. Both systems require the use of a combination of minimum and maximum dose constraints for target volumes, and maximum dose constraints or dose volume constraints for OARs.

For the final dose calculation in XiO the superposition algorithm^(17)^ was used. To ensure a correct calculation of the scatter dose, the manufacturer suggests using 2 mm voxel spacing for the optimization and dose calculation. The optimizer minimizes the cost function F using a conjugate gradient optimization algorithm, which is a special type of gradient descent optimization algorithm.^(18, 19)^ The optimization process ends, after a user defined number of iterations is exceeded or when the cost function converges to the solution. The convergence criterion requires the difference in the cost function for two consecutive iterations to fall below a user selectable threshold. The segmentation of the fluence map into an MLC sequence is only started after the optimization is finished. The segmentation algorithm uses the sliding window technique with the number of intensity levels being selectable by the user. In the last stage, the beam weights are re‐optimized.

KonRad employs a pencil beam algorithm with three decomposed pencil beam kernels for the dose calculation.^(20)^ Tongue and grove effects are accounted for, following the methods described by Webb et al.^(21)^ The optimal voxel spacing according to the manufacturer is $1\sqrt{3}$ times the leaf width. For the Siemens 58 leaf MLC with a leaf size of 1 cm, a dose grid size of 4 mm was employed. The objective function is minimized using a scaled gradient projection algorithm until the difference in the objective function between two consecutive steps is less than 0.1 % or until a finite number (99) of iterations have been performed.

While there are many similarities between the XiO and KonRad TPS in the optimization process, there are important differences as well. Specifically, after optimization constraints or sequencer settings have been changed, KonRad starts the optimization process based on the last iteration prior to the change, whereas XiO resets the fluence map and starts from the beginning. Another important difference is the capability of KonRad to apply the sequencing algorithm for each iteration of the optimization. XiO, on the other hand, only performs the beam segmentation during the final stage of the optimization. Furthermore, KonRad can use a median filter to smooth the fluence map, which reduces the number of beam segments.^(22)^ All KonRad plans discussed in this work use a 2D median filter with a size of 3×3 pixels to remove peaks in the fluence map.

It should be noted that the clinically prescribed objectives do not have the same numerical values as the corresponding parameters for the objectives in the cost function of the treatment system. The clinical objectives were used as a guideline, but during the design of the treatment plans the parameters $D_{\text{max}}$, $D_{\text{min}}$, $\omega_{k}$, and $V_{k}$, and in the case of XiO the power penalties λ and Λ, were tuned individually for each organ and for each of the six plans to obtain the best treatment plan.

Both systems allow the user to choose the number of intensity levels for the discretization of the fluence map needed for the beam segmentation. The number of intensity levels was manually minimized by optimizing and reviewing the plans using different values for that parameter. The plan with the fewest intensity levels and therefore the fewest numbers of segments, which meet all clinical objectives, was selected for further evaluation in this study. The minimization was performed independently for all 6 plans. Dose‐volume histograms (DVHs) for clinical target volume (CTV), planning target volume (PTV) and the relevant OARs as well as dosimetric indices, equivalent uniform dose (EUD), mean dose and maximum dose, were calculated and compared. The KonRad and XiO TPSs do not provide means to calculate EUD, therefore it was calculated with external MatLab programs only after the plans were finished and exported.

Both treatment planning systems allow for the design of verification plans by applying the optimized MLC sequence onto a phantom. A MapCheck device (Sun Nuclear, Melbourne, FL) was used for that purpose. The MapCheck device consists of a 2‐dimensional array of 445 photo diodes housed between two layers of acrylic. Each diode has an active area of 0.8×0.8 mm. The diodes are distributed over a square area of 22×22 cm. The detector spacing is 7 mm in the inner region of the detector and 14 mm in the outer. The device includes a 2 g/cm^3^ layer of acrylic for build‐up and 2 g/cm^3^ for backscatter. Additionally 2 g/cm^3^ of solid water were placed on top of the detector. Therefore, the measurements for dose verification were all done at an effective water equivalent depth of 4 cm, both for 6 and 18 MV photon beams. The dose distribution at 4 cm depth was calculated by the TPS for each field of the six plans and exported to the MapCheck software. The verification plans were generated and the beams from each plan were delivered individually on the MapCheck device using a Siemens Primus linear accelerator. The maximum available dose rate of 300 MU/min for 6 MV and 500 MU/min for 18 MV photon beams was used. The MapCheck software was used to analyze and compare delivered and calculated dose distributions.

## III. RESULTS

**Figure 1:**
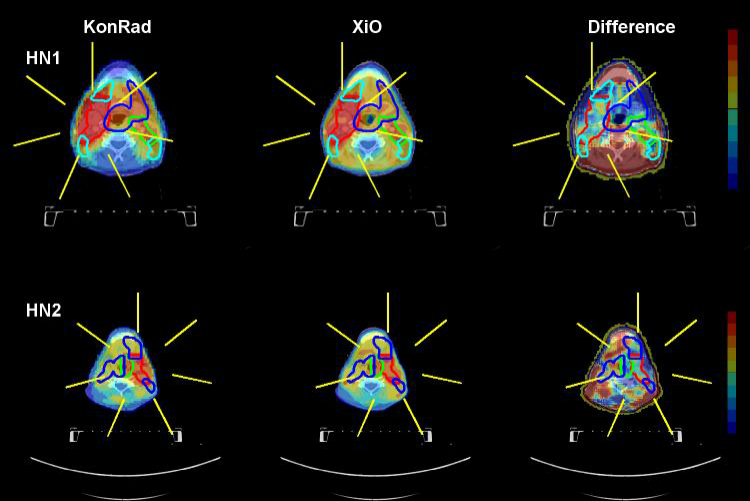
Beam arrangement and dose distribution for cases HN1 (top row) and HN2 (bottom row) from the KonRad (left column) and XiO (middle column) systems. The beam directions are indicated as yellow lines on a transverse CT slice through the isocenter. For HN1 the contours of ${\text{PTV}}_{70}$, ${\text{PTV}}_{65.8}$, ${\text{PTV}}_{63}$, and ${\text{PTV}}_{57.8}$ are shown in red, green, blue, and cyan. For case HN2 the contours of ${\text{PTV}}_{62.4}$, ${\text{PTV}}_{57.6}$, and ${\text{PTV}}_{53.4}$ are shown in red, green, and blue. The dose distribution for the two plans is shown as a color wash overlay. The right column shows dose difference maps. The color wash ranges from +5 Gy in dark red to −5 Gy in dark blue, where positive numbers indicate a higher dose value in the XiO plan.

**Figure 2:**
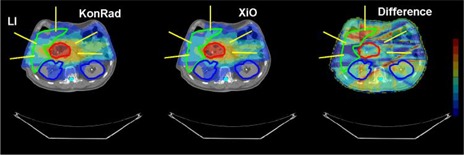
Beam arrangement and dose distribution of the liver case from the KonRad (left) and XiO (middle) systems. The beam directions are indicated as yellow lines on a transverse CT slice through the isocenter. The contours of the PTV is shown in red, the contours of liver, kidneys and spinal cord in green, blue, and cyan, respectively. The dose distribution for the two plans is shown as a color wash overlay. The right panel shows the dose difference between the two plans

**Figure 3:**
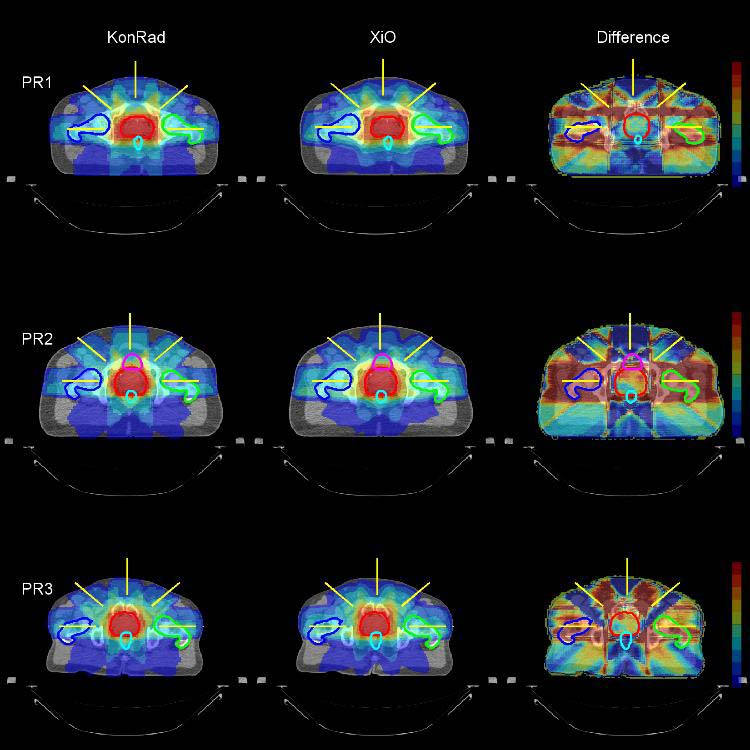
Beam arrangement and dose distribution of the prostate cases PR1, PR2 and PR3 from the KonRad (left column) and XiO (middle column) systems as well as the dose difference between the two plans (right column). The beam directions are indicated as yellow lines on a transverse CT slice through the isocenter. The contours of the PTVs are shown in red, the left and right femoral heads in green and blue, and the rectum in cyan. The dose distribution for the two plans is shown as a color wash overlay. The right column shows the difference of the dose distributions. The color wash for the dose difference ranges from +5 Gy in dark red to −5 Gy in dark blue, where positive numbers indicate a higher dose value in the XiO plan.

Fig. 1 compares the dose distribution obtained from the two systems for the head and neck cases HN1 and HN2. The dose distributions are shown as a color wash overlaid on the transverse CT slice through the isocenter. The results from KonRad are shown in the left column, the results from XiO in the middle column, while dose difference maps are shown in the right column. The beam directions are indicated in yellow. The top row shows the case HN1. The contours of ${\text{PTV}}_{70}$, ${\text{PTV}}_{65.8}$, ${\text{PTV}}_{63}$, and ${\text{PTV}}_{57.8}$ are shown in red, green, blue, and cyan, respectively. The dose distributions from both TPS are similar with minor differences. Qualitatively both plans achieve similar coverage of the PTVs. The XiO plan appears to be slightly more conformal. On the other hand the dose distribution generated by the KonRad plan yields lower doses to unspecified tissue and OARs located away from the PTVs. Qualitatively such results were similar in the second H&N case (second row), in the liver case (Fig. 2) and in the three prostate cases (Fig. 3). The dose difference maps of cases LI, and PR1–3 demonstrate that although the integral dose distribution is very similar, the relative beam weights of the five fields can be different depending on the treatment planning system.

**Figure 4:**
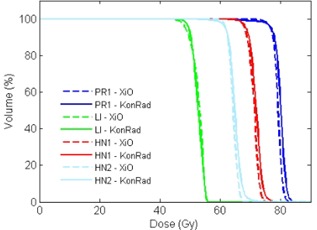
Comparison of the DVH for the PTV from the KonRad (solid) and XiO (dashed) systems. The prostate example PR1 is shown in blue, the head and neck cases HN1 and HN2 in red and light blue, and the liver case in green. Besides a slightly steeper dose fall‐off of the XiO plans, the DVHs are similar.

A comparison of the DVHs yields a more quantitative result than the qualitative comparison of the dose distribution itself. Fig. 4 shows the DVHs for the PTV for cases HN1, HN2, LI and PR1 for both TPS. The DVHs for cases PR2 and PR3 would look similar to PR1. The KonRad results are displayed as solid lines, the XiO results as dashed lines. The prostate case PR1 is shown in blue, the liver case LI in green. For the head and neck case HN1 the DVH for ${\text{PTV}}_{70}$ is shown in red, for case HN2 the DVH for ${\text{PTV}}_{62.4}$ is shown in light blue. The XiO and KonRad plans achieved similar PTV coverage, exceeding the prescribed requirements on the dose coverage in each case. In all three cases, a slightly sharper dose fall‐off of the XiO plans was noticed.

Table 1 shows the prescription dose, mean dose $D_{\text{mean}}$, the dose received by 98% of the volume $D_{98}$, the equivalent uniform dose EUD and maximum dose $D_{\text{max}}$ to the PTVs for the XiO and KonRad plans. For the prostate and the liver cases the values for $D_{\text{mean}}$, $D_{\text{max}}$ and EUD are very similar between the two systems, the differences being less than 1.0 Gy. The head and neck cases with different PTVs posed a larger challenge for both treatment planning systems. Still the mean doses to the PTVs differed by 1.1 Gy at most, with KonRad generating the higher values. This is also reflected in the higher values for $D_{\text{max}}$ of the KonRad plans.

**Table 1:** Comparison of the RT plans generated by the KonRad and XiO treatment planning systems and dosimetric outcome for the PTV. The three prostate and the liver cases had one PTV each, whereas in the head and neck examples several PTVs with different prescription doses were defined.

*Case ID Tumor Site*	*TPS*	*Prescribed Dose (Gy)*	*Mean Dose (Gy)*	*D98 (Gy)*	*EUD (Gy)*	*Maximum Dose (Gy)*
HN1	KonRad	70.0	71.7	67.2	70.5	77.9
Head&Neck		65.8	66.5	59.7	62.2	72.9
		63.0	64.6	56.4	53.4	76.1
		57.8	59.3	52.4	25.1	74.9
	XiO	70.0	71.0	67.0	70.7	75.7
		65.8	66.2	63.1	65.6	70.9
		63.0	63.5	50.5	29.0	71.5
		57.8	60.3	55.8	43.4	72.9
HN2	KonRad	62.4	64.7	60.1	62.4	75.5
Head&Neck		57.6	60.9	57.0	60.1	66.3
		53.4	56.1	51.3	48.87	67.3
	XiO	62.4	63.9	61.0	63.7	68.7
		57.6	60.1	57.0	59.9	64.9
		53.4	55.5	51.3	54.7	66.7
LI1	KonRad	50.4	52.5	48.6	51.8	56.1
Liver	XiO	50.4	52.5	47.2	51.5	56.5
PR1	KonRad	77.4	79.7	74.7	77.2	84.5
Prostate	XiO	77.4	79.0	75.3	78.4	83.9
PR2	KonRad	77.4	79.4	76.2	79.0	82.9
Prostate	XiO	77.4	80.4	77.1	80.0	83.5
PR3	KonRad	77.4	80.0	76.9	79.7	83.5
Prostate	XiO	77.4	81.0	77.7	80.6	84.5

For the liver and prostate plans, as well as for the PTVs with the two highest prescription doses in the head and neck examples, the EUD is almost as high as $D_{\text{mean}}$ and both values are exceeding the prescription dose. This again illustrates that the plans from both systems achieve similar and good coverage. For cases HN1 and HN2, neither system could achieve as good coverage for the PTVs with the lower prescription doses compared to the primary PTV which was deemed more important by the physician. This is most obviously reflected in the EUD values for ${\text{PTV}}_{63}$ and ${\text{PTV}}_{57.8}$ for HN1 and ${\text{PTV}}_{53.4}$ for HN2, which are below the prescription doses. In these two examples no plan could be found which would have provided better coverage of these targets without compromising the primary PTV coverage.

**Figure 5:**
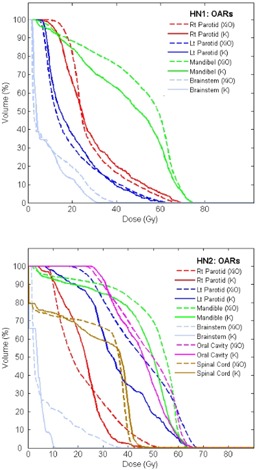
Comparison of DVHs for the relevant OARs of the cases HN1 (top) and HN2 (bottom). XiO plans are shown as dashed and KonRad as solid curves.

Whereas the PTV coverage was very similar, the DVHs for OARs differed between the plans generated by the two systems. The upper panel of Fig. 5 shows DVHs for the left and right parotid, the mandible and the brainstem for patient HN1. The KonRad plan resulted in a better sparing of the mandible and the brainstem, whereas the XiO plans achieved a better sparing of the left parotid. The a smaller partial volume of the right parotid received doses above 25 Gy using the XiO plan, but a larger partial volume received doses between 10 and 15 Gy. For patient HN2 the DVHs for the relevant OARs are shown in the bottom part of Fig. 5. In this case the KonRad plan showed improved sparing of all OARs.

**Figure 6:**
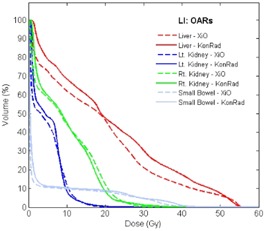
Comparison of DVHs for four OARs (liver, right and left kidneys, and small bowel) of the liver patient. XiO plans are shown as dashed and KonRad as solid curves.

Fig. 6 shows a similar comparison of liver, kidney, and small bowel DVHs for the patient with liver cancer. The DVHs for both kidneys and for the small bowel only show minor differences, but the XiO plan achieved a slightly better sparing of the liver.

**Figure 7:**
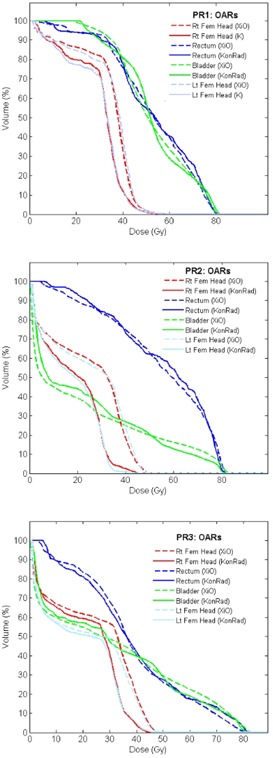
Comparison of DVHs for four OARs (right and left femoral head, rectum, and bladder) of the prostate examples PR1 (top), PR2 (middle) and PR3 (bottom). XiO plans are shown as dashed and KonRad as solid curves.

The DVHs of left and right femoral heads, of the bladder and of the rectum for the prostate patients PR1, PR2 and PR3 are shown Fig. 7. The DVHs from the XiO plan are shown dashed, the ones from KonRad as solid lines. Concerning the femoral heads, KonRad achieved a superior sparing. The DVHs for rectum and bladder are clinically equivalent for both systems. For example for case PR1 the mean dose to the bladder is 52.9 Gy for both the XiO and the KonRad plan. The mean dose of the rectum is 52.7 Gy for the XiO plan compared to 52.9 Gy for the KonRad plan.

The results of the beam weight and beam segmentation optimization are shown in Table 2. The plans optimized using KonRad resulted in a reduced number of MUs (between 18% and 35%). With the exception of the first head and neck case (HN1), KonRad required fewer number of intensity levels than XiO. In all cases, the number of beam segments was reduced between 10% and 50% for the KonRad plans. The reduction of both the number of MUs and the number of segments led to shorter delivery times. The time savings of the KonRad plans ranged from 1.5 min for the third prostate case to over 5 minutes for case HN1. On average the time saving was 3.5 min or 28.6%. Table 3 shows the average number of MUs per segment, the number of MUs for the longest and shortest segments, as well as the size of the smallest and largest segment for the six plans. The most significant difference between the two systems was that the XiO plans consistently included segments with very small field sizes $\left(<1.5{\text{cm}}^{2}\right)$.

**Table 2:** Comparison of the segmentation results and treatment times for KonRad and XiO treatment plans.

*Tumor Site*	*TPS*	*MU/fx*	*Total Segments*	*Intensity Levels*	*Duration(sec)*
HN1	KonRad	788	117	10	966
	XiO	1032	128	8	1283
HN2	KonRad	727	85	6	823
	XiO	883	106	7	1008
LI1	KonRad	311	52	10	475
	XiO	436	69	11	607
PR 1	KonRad	348	49	10	453
	XiO	535	82	12	724
PR2	KonRad	339	37	10	301
	XiO	486	76	12	593
PR3	KonRad	314	45	10	361
	XiO	427	55	9	452

**Table 3:** Detailed comparison of the segmentation results between the KonRad and the XiO systems for the six cases.

*Tumor Site*	*TPS*	*MU/Segment*	*Shortest Segment (MU)*	*Longest Segment(MU)*	*Smallest Segment(cm^2^)*	*Largest Segment (cm^2^)*
HN1	KonRad	6.7	4.0	24.0	2.0	15.1
	XiO	8.1	5.0	23.0	1.2	14.1
HN2	KonRad	8.6	6.8	10.4	2.0	13.1
	XiO	8.3	5.6	21.4	1.1	12.8
LI1	KonRad	6.0	5.0	15.0	1.6	9.3
	XiO	6.3	5.0	13.0	1.0	8.8
PR1	KonRad	7.1	5.0	29.0	2.4	11.0
	XiO	6.5	5.0	15.0	1.0	10.7
PR2	KonRad	9.2	5.4	33.8	2.4	7.9
	XiO	6.4	4.9	13.5	1.0	7.9
PR3	KonRad	7.0	5.2	24.5	2.0	8.5
	XiO	7.8	5.7	18.0	1.5	9.2

**Figure 8:**
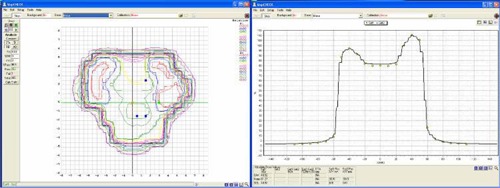
Dose verification of the AP field for the prostate case. The left panel shows the dose distribution (isodose lines) calculated by the KonRad TPS together with the ones measured with the MapCheck device. The right panel shows the vertical profile of the dose distribution. The measured dose points are shown as yellow circles, the calculated dose profile as a solid line. For this field 202 out of 205 points passed the 3%/3mm acceptance criteria.

The twelve plans generated by the two treatment planning systems were all deliverable. For the QA of the IMRT treatment plans, a coronal dose distribution at a depth of 4 cm was measured for each field separately. With an acceptance criteria of 3% dose difference, 3 mm distance‐to‐agreement and a 10% threshold per data point, the passing rate for all fields of all plans always exceeded 90%. The passing rate for the KonRad plans was on average 3.1 percent points better than for the corresponding XiO plan. Table 4 summarizes the results of the MapCheck analysis. Fig. 8 shows the result for the AP field of the KonRad plan for the prostate case. On the left side the overlaid calculated and measured isodose lines are shown, and on the right side a profile of the dose distribution along the center of the field is shown, with the measured data points (circles) and the calculation (solid line).

**Table 4:** Results of the dose verification of the twelve plans on the Sun Nuclear MapCheck device. The acceptance criteria demanded a dose difference less than 3% within 3 mm. With passing rates well above 90%, all plans were clinically acceptable.

*Tumor Site*	*TPS*	*Dose Points pass*	*Dose Points failed*	*Passing Rate*
HN1	KonRad	1721	46	97.4%
	XiO	1400	137	91.1%
HN2	KonRad	1772	83	95.5%
	XiO	1883	187	91.0%
LI1	KonRad	591	34	94.6%
	XiO	539	42	92.8%
PR1	KonRad	895	6	99.3%
	XiO	859	26	97.1%
PR2	KonRad	606	2	99.7%
	XiO	649	16	97.6%
PR3	KonRad	670	3	99.6%
	XiO	771	17	97.8%

## IV. DISCUSSION

For all six cases both the plans from KonRad and from XiO were determined to be clinically acceptable. The plans from the two different systems generated equivalent dose distributions. Especially the PTV coverage was very similar, which is reflected in similar values for the mean dose for the PTV. However differences concerning the PTV coverage between the two systems were noticed, although none of them was judged to be clinically significant: the DVHs for the PTVs of the XiO plans displayed a sharper dose fall‐off and the maximum dose to the PTV was slightly higher using the KonRad plans. This was not caused by the reduced numbers of intensity levels in KonRad. Even increasing this parameter during optimization did not improve the dose fall‐off or reduce the maximum dose. Potentially the smoothing of the fluence map with a median filter as well as the slightly larger voxel size within KonRad are possible explanations. However, since the difference was not clinically important, we did not explore this topic any further.

Both systems achieve good target coverage for the primary target, however for the two more complicated head and neck cases HN1 and HN2 with multiple distinct targets of different prescription doses, neither system was able to achieve good coverage for the lower priority targets (${\text{PTV}}_{63}$ and ${\text{PTV}}_{57.8}$ for HN1; ${\text{PTV}}_{53.4}$ for HN2), which is reflected in the low EUD values, compared to the mean dose and prescription dose. It should be pointed out, that the EUD values were not available during the design of the treatment plan, because it can not be calculated by KonRad and XiO. Therefore, the quality of the coverage was judged based on the DVH, $D_{\text{mean}}$ and $D_{98}$.

The dose distribution outside the target volume of both systems differed in details; however by slight adjustments to the optimization constraints the differences were minimized. Generally, it was observed that KonRad achieved superior sparing of OARs located several cm away from the PTV. The femoral heads of the prostate patient are the prime example for this observation. On the other hand, for OAR in close proximity to the tumor, XiO is able to achieve a better sparing, as illustrated by the DVH of the liver in the case of the patient treated for liver cancer (case LI), where the PTV is embedded in the liver.

In all six examples the segmentation using KonRad was more efficient, resulting in fewer segments, fewer MUs and therefore shorter delivery times by up to 5 min, while achieving similar coverage of the PTV and a clinically equivalent sparing of the OARs. Unlike KonRad, XiO allows the user to manually change or remove segments after the optimization. Segments with small field sizes and small number of MUs could in principle be removed this way. However, because this method is labor intensive and because the intention of this work was to evaluate the implemented segmentation algorithm, such modifications were not included in our analysis. It was already reported^23^, ^24^ that the segmentation algorithm^(11)^ implemented in KonRad, is one of the most efficient methods concerning the number of MUs. However, concerning the number of beam segments no such observation was made previously. Furthermore, segmentation algorithms in both systems rely on similar techniques. Therefore, the smaller numbers of beam segments using KonRad must probably be attributed in some extend to the smoothing of the fluence map before converting it into a MLC leaf sequence.

The two systems employ different dose calculation engines. The pencil beam algorithm of KonRad is faster than the XiO superposition algorithm. This advantage is enhanced by the larger voxel size needed with KonRad for a Siemens 58 leave MLC, reducing the computing time even further. The dose calculation for the verification plans on a MapCheck device for both systems shows clinically fully satisfactory agreement between the calculated and the observed dose distributions. However it has to be noted that this method of evaluating the quality of the dose calculation is limited, because the MapCheck device basically represents a homogenous block of acrylic. Therefore, using the MapCheck system for the dose verification does not simulate an environment where inhomogeneity corrections would play an important role.

## V. CONCLUSION

Treatment plans generated using the new Siemens KonRad treatment planning system were dosimetrically equivalent to plans generated with the established XiO system. The sequencing algorithm of KonRad was more efficient, reducing the number of beam segments and therefore delivery time on average by more than 25%. The dose verification with the MapCheck device did not reveal any significant difference in the quality of the plans concerning accuracy of the dose calculation or deliverability of the fields.

## ACKNOWLEDGEMENT

This work was partially supported by Siemens Medical Solutions.
